# Effect of grand multiparity on adverse maternal outcomes: A prospective cohort study

**DOI:** 10.3389/fpubh.2022.959633

**Published:** 2022-10-13

**Authors:** Tamirat Tesfaye Dasa, Michael A. Okunlola, Yadeta Dessie

**Affiliations:** ^1^Life and Earth Sciences Institute, (Including Agriculture and Health) Pan African University, Ibadan, Nigeria; ^2^Midwifery Department, College of Medicine and Health Sciences, Hawassa University, Hawassa, Ethiopia; ^3^Department of Obstetrics and Gynecology, College of Medicine, University College Hospital, University of Ibadan, Ibadan, Nigeria; ^4^School of Public Health, College of Health and Medical Sciences, Haramaya University, Harar, Ethiopia

**Keywords:** maternal complication, parity, obstetrics, follow up, Ethiopia, cohort study

## Abstract

**Background:**

Grand multiparity remains a risk factor for a wide range of obstetric complications, especially in developing countries. Grand multiparity has been shown to increase the risks of medical and obstetric complications during pregnancies. However, in a research setting, the risk factors associated with adverse maternal outcomes have yet to be adequately investigated among grand multiparity. Furthermore, there is limited information that examines the effect of grand multiparity on pregnancy outcomes in Ethiopia through prospective follow-up design.

**Objective:**

This study aimed to investigate the effect of grand multiparity on pregnancy outcomes in selected public hospitals in the Sidama Region State of Ethiopia.

**Methods:**

A prospective cohort study design was employed on 837 pregnant women who were admitted for delivery in selected public hospitals from January 1 to August 31, 2021. The study subjects were recruited during admission for labor and delivery. Every woman who was admitted to labor wards was screened for eligibility. The exposed group in this cohort was grand multiparity, and the non-exposed group was multiparity. Data collection was started from the first contact after admission and follow-up to discharge for adverse maternal outcomes. The risk factors for adverse maternal outcomes in grand multiparity were investigated using multivariable Poisson regression analysis. The risk factor was reported as an adjusted risk ratio (ARR) with a 95% confidence interval (CI). When the *P*-value was <0.05, statistical significance was declared.

**Results:**

The cohort's overall cumulative incidence of adverse maternal outcomes were 39.9% (95%CI: 36.6, 43.4%). Among exposed groups, the incidence of adverse maternal outcomes were 47.1% (95%CI: 41.0–53.2) and 36.3% (95% CI: 32.3–40.6) the multiparity. When compared to multiparous women, grand multiparity was associated with a greater risk of postpartum hemorrhage (ARR = 2.1; 95%CI:1.6–2.7) and malpresentation (ARR = 1.3; 95% CI: 1.01–1.7).

**Conclusions:**

Pregnant women with grand multiparity have a higher incidence of adverse maternal outcomes. Grand multiparity increased the risk of adverse maternal outcomes such as postpartum bleeding and malpresentation. In low-resource settings, we recommend that community health education, the provision of accessible and effective contraceptive services, and increased awareness of the adverse maternal outcome among grand multiparity during pregnancy on obstetric performance should be prioritized. Also, trained health providers can effectively decrease the risk factor with good antenatal care and delivery.

## Introduction

A grand multiparous (GMP) woman, according to the International Federation of Obstetrics and Gynecology, has had at least five to nine prior term deliveries, and this classification has recently been widely recognized by many authors (1–4). In Ethiopia, grand multiparity is defined as a woman who has had five or more previous deliveries after the second trimester of pregnancy (5, 6).

Grand multiparity is becoming less of a concern in many developed countries, with a low prevalence of 2–4 % of all births due to limiting birth, birth planning, and the widespread use of modern contraceptive technologies. Whereas, in developing countries, grand multiparity remains a major concern, with a high prevalence of childbearing at 19 % (7–9). Similarly, studies show that Sub-Saharan African countries have the highest rate of grand multiparity, with incidence rates ranging from 18 to 28 % in some countries (3, 10, 11). According to Ethiopia's demographic health and survey report of 2016, the prevalence of grand multiparity was 26 percent (12).

Grand multiparity is one of the leading causes of death and disability among women of reproductive age in low and middle-income countries, and it is related to problems during pregnancy and childbirth (13, 14). It has also been suggested that it is a distinct risk factor for several maternal problems (7). Particularly, grand multiparity has been linked to poor maternal outcomes during labor and delivery in underdeveloped nations, including an increased risk of postpartum hemorrhage, cesarean section, and malpresentation (15–17).

Grand multiparity has been demonstrated to increase the risk of medical and obstetric problems (6, 18–20). As previously reported, grand multiparity is related to fewer adverse maternal outcomes than multiparity (4, 5, 10, 18, 21–30). Still, some other studies conducted in developing countries indicated a non-significant difference between adverse maternal outcomes among grand multiparity and multiparity (1, 3, 6, 19, 31–35). There has been inconclusive evidence on the effect of grand multiparity on adverse maternal outcomes based on the data of various studies. Furthermore, in the study setting, the association between grand multiparity and possible poor maternal outcomes is not adequately identified. As a result, the objective of this study was to examine the risk of unfavorable maternal outcomes among grand multiparous women in the Sidama National Regional State of Ethiopia.

## Methods and materials

### Study areas, design, and time frame

The research was carried out in Ethiopia's Sidama Regional State. It is located in the country's southern region, 273 kilometers south of Addis Ababa, Ethiopia's capital city. The region is divided into 36 districts (6 urban and 30 rural), with Hawassa as the administrative center. The capital of the region is Hawassa. The Oromia region with the Gedeo Zone borders it on the south, the Wolaita Zone on the west, and the Oromia region on the north and east. The Sidama has an estimated population of 8.8 million people (4.01 percent of the national population). There are 123 primary health center units and 17 hospitals in the region (One Comprehensive Referral hospital, four General hospitals, and 12 Primary Hospitals).

The prospective cohort study was carried out at five government hospitals that are designated as general and referral hospitals, meaning they provide specialist care for both the mother and the newborn. The study took place between January 1 and August 31, 2021.

### Cohort choice, enrollment, and exclusions

The region's multiparous and grand multiparous women served as a source population. The exposed group consisted of women with grand multiparity (Para 5-9) who attended the selected hospitals. As a result, during the study period, all women were admitted to the participating hospitals for birth. Multiparous women (Para 2–4) were included as a non-exposed group. The controls were chosen based on the five age-interval groups and interviewed of them on the same day as the exposed group. Women with multiple pregnancies and chronic illnesses related to pregnancy were more occurred adverse maternal outcomes among these groups due to these reasons were excluded. Also, women who are mentally or critically ill and unable to communicate were excluded from the study.

### The size of the sample and sampling technique

To estimate the sample size, the sample size was computed using a double population proportion formula with continuity correction using EPI Info version 7 STAT CALC software cohort research as specified by Fleiss (36). The maximum sample was computed using the following assumptions: a two-sided confidence level of 95 percent, a power level of 80 percent, r = the ratio of exposed to unexposed group 1 to 2, and p1 = the incidence of preterm outcome among women with grand multiparity of 35 percent (30), p2 = incidence of preterm outcome among multiparity 24% (34). Because we couldn't discover any similar research in Ethiopia, we used data from a study completed in Pakistan. Adding a 10 % loss follow-up for this study. Finally, 837 people were enrolled in the study (279 grand multiparity and 558 multiparity). There are 17 hospitals in the Sidama region (12 primary hospitals, four general hospitals, and one comprehensive referral hospital). Five hospitals (30%) out of a total of 17 governmental hospitals were chosen. Those hospitals were chosen based on the given full package of maternal and newborn services. Using the likelihood proportional to sample size and hospital customers' follow-up from last year's delivery admission reported, an appropriate sample size was assigned to each hospital. The participants in the study were sampled study participants by using systematic sampling until the desired sample size was obtained.

### Tools and procedures for data collection

The data gathering tools were created in the English language and subsequently translated by language experts into the local language (Amharic). Following a thorough assessment of various types of published literature, the questionnaires were developed (6, 12, 17, 18, 20, 23–25, 32–35, 37, 38). The client's information, socio-demographic factors, obstetrics, and reproductive health history characteristics, maternal health services used during current and previous pregnancies, nutritional status of women, and adverse maternal outcomes parts were all included in the interview questionnaires of participants. The information was gathered from the time of admission to and delivery to the maternal wards. As data collectors and supervisors, qualified and trained health professionals were sought. Before beginning the actual data collection, 5 days of training were conducted for the data collectors and supervisors. Following the training, a pre-test was conducted on 5 % of the sample size outside of the actual data collection site to guarantee tool consistency.

### Operational definitions

#### Adverse maternal outcome

The presence of one or more of the following characteristics: Pregnancy-induced hypertension, premature membrane rupture, malpresentation, cesarean section, and obstetric hemorrhage from hospital admission to discharge.

#### Grand multipara

A woman who has already given birth to five to nine newborns at a gestational age of 28 weeks or more (39, 40).

#### Adverse maternal outcome

The presence of one or more of the following characteristics: Pregnancy-induced hypertension, premature membrane rupture, malpresentation, cesarean section, and obstetric hemorrhage from hospital admission to discharge.

Cesarean delivery is defined as the delivery of a fetus via a surgical procedure involving the abdominal and uterine walls (41).

Pregnancy-induced hypertension is defined as blood pressure greater than or equal to 140/90 mmHg, taken after a period of rest on two occasions 4 hours apart, or pressure ≥160/110 mmHg on one occasion in a previously normotensive woman (42, 43).

Malpresentations are all presentations of the fetus part that first enters the maternal pelvis Other than the vertex. For example, “the most common malpresentation is the breech presentation (44).

Postpartum hemorrhage is commonly defined as blood loss that exceeds 500 milliliters (mL) after vaginal birth and 1,000 mL after a cesarean section (45, 46).

A short birth interval was defined as a period of fewer than 33 months between two consecutive live births (33 months = 24 months from birth to conception period + 9 months pregnancy duration) (47).

### Variables and measures that affect the outcome

The major outcome of interest was adverse maternal outcomes during pregnancy among grand multiparity.

### Variables that potential confounding

Various risk factors, including maternal characteristics and potential confounding variables, were gathered at baseline and afterward on several risk factors during labor and delivery. A complete pregnancy history, including pre-existing medical issues, was also obtained. Socio-demographic characteristics such as maternal age, religion, ethnicity, household wealth status, respondents' education, husband's education, and occupation; obstetrics and some medical factors such as the history of preterm birth, history of stillbirth, and unplanned pregnancy were included as potential confounding variables. MUAC (Middle-Upper Arm Circumference), BMI (Body Mass Index), and infant sex.

### Data management and analysis

Epi Data version 3.02 software was used to enter double data. For analysis, the entered data was exported to STATA 14 version software. The study population was described using descriptive statistics such as a frequency table and summary indices. For continuous variables, we used visual inspection and statistical tests such as Shapiro-Wilk tests to see whether they were regularly distributed, and the variable was regarded to be so if the *p*-value was >0.05. It was necessary to evaluate risk using a model designed for uncommon outcomes. As a result, Generalized Linear Model (GLM) with an identity log and binomial link function to calculate relative risk (log-binomial) was used. There is a difficulty with convergence while using the log-binomial approach, hence a modified Poisson regression was switched. Improved precision for relative risk estimation and robustness to omitted covariates are two advantages of adopting the modified Poisson regression model. In a bivariable study with a *p*-value of 0.2, all variables risk factors with outcomes of interest were selected candidates for the multivariable Poisson regression model. Multivariable Poisson regression analysis was used to evaluate the risk factors associated with grand multiparity and adverse maternal outcomes in the final model. The Hosmer-Lemeshow goodness-of-fit tests were used to determine model fitness. The regression model's risk factors for grand multiparity were presented using an adjusted risk ratio (ARR) with a 95% CI. When the *P*-value < 0.05, statistical significance was declared. At a cut-off point of 10, the Variance Inflation Factor (VIF) was used to ensure the presence of multicollinearity among explanatory variables (48) and there has been no multicollinearity in the data.

### Study ethical considerations

The study was submitted for clearance to the Pan Africa University Life and Earth Sciences Institute, the University of Ibadan, and the University of Ibadan/University College Hospital, Ibadan Ethics Committee (Ethics committee assigned number /EC/20/0439). Following the approval, concerned bodies in the data gathering zones received an official letter of cooperation. The Sidama Regional Health Bureau gave their permission. Before any data collection processes, each study participant signed a written informed consent form or some of the used figure prints after giving volunteer consent to those who were unable to write and read. After discussing the study's goals, data collection processes, the benefits, and hazards of participating in the study, and the study subjects' voluntariness, consent was acquired. The study follows the Declaration of Helsinki's guidelines (49).

## Results

### Participants' baseline characteristics

**Figure 1:**
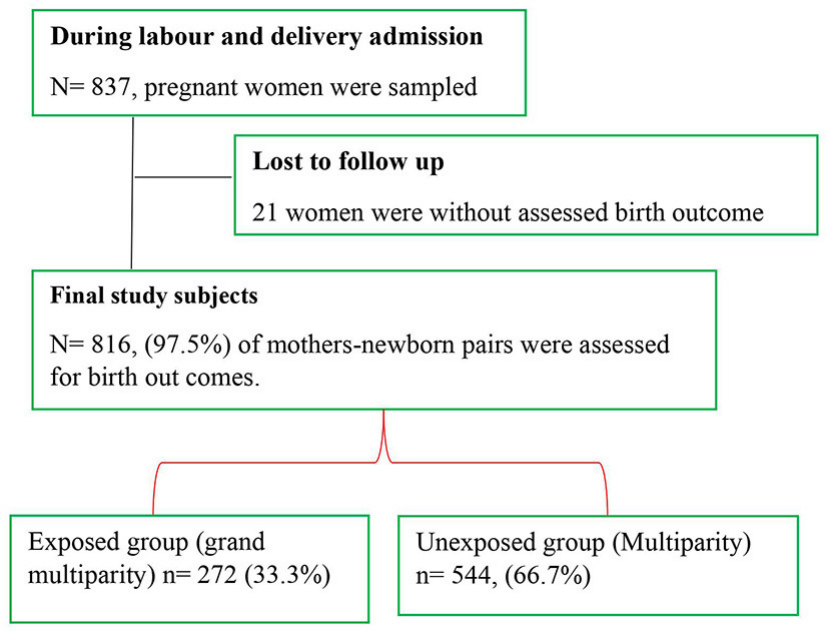
Flowchart of study participants in Ethiopia's Sidama Region's five governmental hospitals in 2021.

The study enrolled a total of 837 pregnant women (558 multiparous and 279 grand multiparous), with 816 of them being followed up on maternal outcomes. The response rate was 97.5%. Around 21 study participants were excluded from the analysis due to refusal at the beginning and missing appropriate birth outcome data (Figure 1). Slightly more than two-thirds (67.6%) were aged between 24 and 34 years with (38.5%) living in rural areas. The participants lacked formal education (31.4%) and were mostly housewives (67.3%). Women's husbands had completed secondary school (28.6%) and were merchants (29.9 %). During pregnancy, around (70.1%) of study participants received dietary nutrition counseling. Three-fourths (75.0 %) did not have male involvement during their most recent pregnancy follow-up. Half of the study groups (50.3%) had a short birth interval. The majority (93.4%) of the study participants had ANC follow-up for current pregnancy and about one-third started their first ANC booking before 16 weeks of gestational age (30.7%) (Table 1).

**Table 1:** Selected baseline sociodemographic and reproductive health characteristics of the study groups in public hospitals in the Sidama Reginal State of Ethiopia, 2021.

**Variables**	**Category**	**Frequency (No_)**	**Percent (%)**	***P*-value**
Age	18–24 24–34 35–44	120 552 144	14.71 67.65 17.65	*P* < 0.001
Residence	Urban Rural	502 314	61.52 38.48	*P* < 0.001
Educational statues	Lack of formal education Elementary school Secondary school Tertiary and above	256 207 215 138	31.37 25.37 26.35 16.91	*P* < 0.001
Occupation status	Housewife Merchant Government employee Daily laborer	549 101 118 48	67.28 12.38 14.46 5.88	*P* < 0.001
Family monthly income	Low Middle High	277 362 164	34.50 45.08 20.42	P=0.025
Husband occupation	Daily laborer Farmer Merchant Government employee	150 201 244 220	18.40 24.66 29.94 26.99	*P* < 0.001
Husband Education	Lack of formal education Elementary school Secondary school Tertiary and above	135 221 233 225	16.58 27.15 28.62 27.64	*P* < 0.001
Dietary nutrition counseling	No Yes	240 563	29.89 70.11	*P* = 0.259
Male involvement during pregnancy	No Yes	612 204	75.00 25.00	*P* < 0.001
Age at first marriage	Age < 18 years Age ≥18 years	310 504	38.08 61.92	*P* < 0.001
Birth interval	= < 36 months > 36 months	410 406	50.3 49.7	*P* = 0.4
Pregnancy planned	No Yes	163 652	20.0 80.0	*P* < 0.001
ANC follow-up current pregnancy	No Yes	54 762	6.6 93.4	*P* < 0.001
First booking GA for ANC	< = 16weeks of GA > 16 weeks of GA	234 528	30.7 69.3	*P* < 0.002
Number of ANC visit	1–3 4 ^**+**^	462 296	60.9 39.1	*P* < 0.002
Knowledge of danger sign	No Yes	365 451	44.7 55.3	*P* = 0.065

### Adverse maternal outcome characteristics

About (20.1%) were delivered via cesarean section, with (12.5%) suffering from malpresentation. More than half (54.5 %) of the study population had anemia, and one out of twenty women (4.9%) had a postpartum hemorrhage complication. Premature rupture of the membrane was observed in 11.2% of the sampled population. Preeclampsia affected about one out of every eight women (12.9%) and eclampsia affected 2.9% of the studied population (Table 2).

**Table 2:** Adverse maternal outcome characteristics in five governmental hospitals in Ethiopia's Sidama regional state in 2021. *N* = 816.

**Characteristics**	**Category**	**Frequency (No_)**	**Percent (%)**
Cesarean delivery	No Yes	649 167	79.5 20.5
Malpresentation	No Yes	714 102	87.5 12.5
PROM	No Yes	725 91	88.8 11.2
Women undergo induction labor	No Yes	750 66	91.9 8.1
Postpartum hemorrhage	No Yes	776 40	95.1 4.9
Preeclampsia	No Yes	748 68	91.7 8.3
Eclampsia	No Yes	792 24	97.1 2.9
Anemia	No Yes Missing	334 445 37	40.8 54.5 4.5
Total stay in hospitals	Discharge in 24 hrs Discharge in 72 hrs Discharge more than 72 hrs	437 272 107	53.6 33.3 13.1

### Incidence of adverse maternal outcome

**Figure 2:**
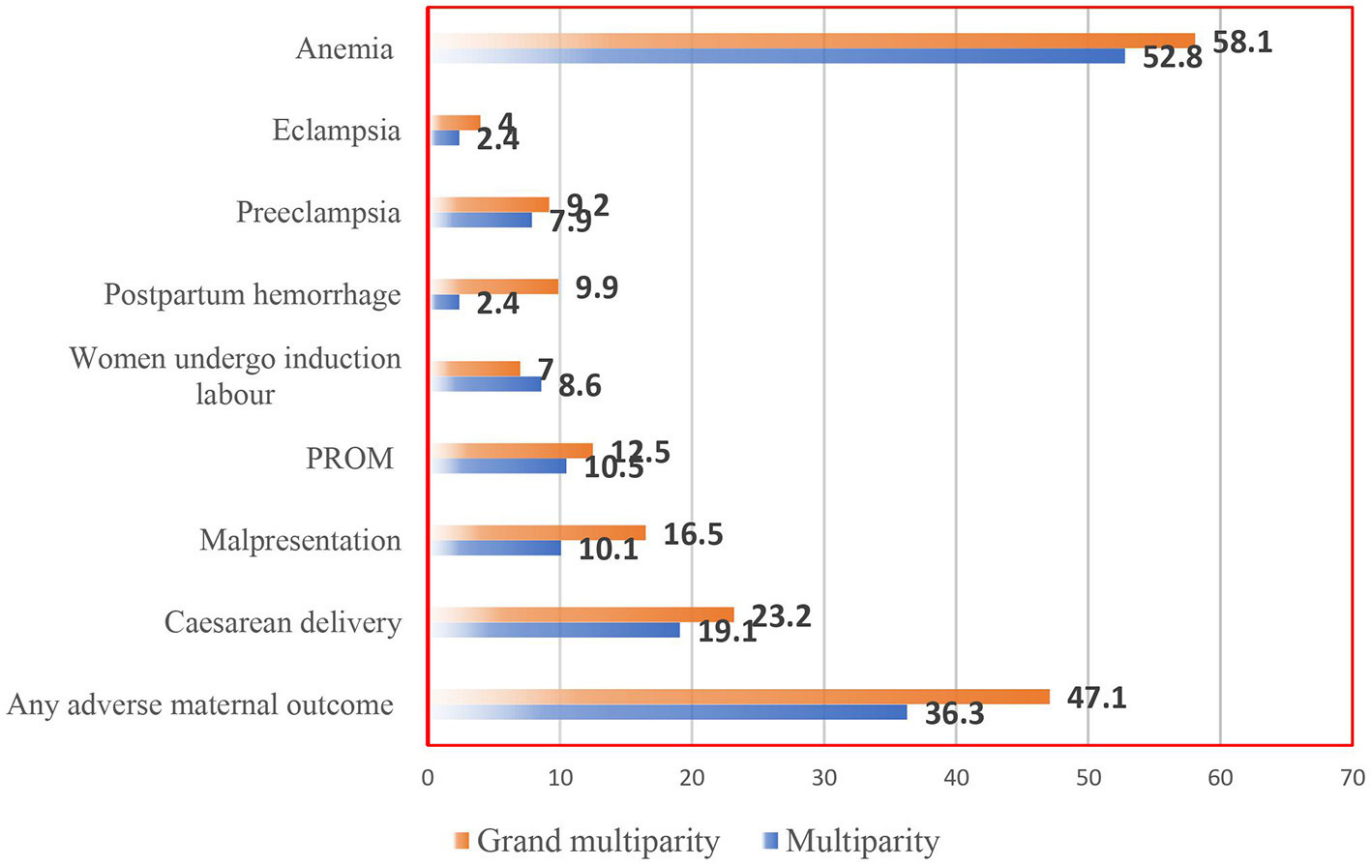
Incidence of unfavorable maternity outcomes in five governmental hospitals in Ethiopia's Sidama Regional State, 2021.

Of the studied subjects, the total occurrence of adverse maternal outcomes was 39.9% (95% CI: 36.6, 43.4%) in the cohort. Among the grand multiparity group, the rate of adverse maternal outcomes was 47.1% (95 % CI: 41.0–53.2) and 36.3% (95% CI: 32.3–40.6) among the multiparous women. Thus, in grand multiparity the rate of cesarean was 23 (95% CI: 18.3–28.6) per 100 pregnancies, malpresentation was 16.5 (95 % CI: 12.3–21.5) per 100 delivering women, postpartum hemorrhage was 9.9 (95% CI: 6.6–14.1) per 100 deliveries. The percentages of multiparity vs. grand multiparity for each adverse maternal outcome are shown in the figure details below (Figure 2).

### Compared incidence of occurrence among parity and adverse maternal outcome

The total incidence of grand multiparous related to multiparous in a meaningful way (39.3 % vs. 60.7%, *p*-value< 0.003). The incidence of postpartum hemorrhage was higher among high parous than low parous women (67.5 vs. 32.5%, *P* < 0.001). Cesarean deliveries were less common in high parous women than in low parous women (37.7 vs. 62.3 %, *p*-value <0.177), but this was not statistically significant. Almost close to half (44.1%) of grand multiparous and more than half (55.9%) of multiparous women had malpresentation during delivery, with a *p*-value<0.014 (Table 3).

**Table 3:** In the Sidama Regional State of Ethiopia, 2021, the result of a modifier poison regression model to the probability of an adverse maternal outcome for grand multiparity.

**Adverse maternal outcome**	**Category**	**Multiparity No_ (%)**	**Grand multiparity No_ (%)**	**CRR (95% CI)**	**ARR (95% CI)**
Cesarean delivery	No Yes	440 (80.8) 104(19.1)	209(76.8) 63(23.2)	Ref. 1.2(0.9–1.46)	Ref 1.07(0.8–1.4)
Malpresentation	No Yes	487(89.5) 57(10.5)	227(83.5) 45(16.5)	Ref. 1.4(1.1–1.8) **	Ref 1.3(1.01–1.7) *
PROM	No Yes	487(89.5) 57(10.5)	238(87.5) 34(12.5)	Ref. 1.1(0.9–1.5)	Ref. 1.1 (0.8–1.5)
Women undergo induction labor	No Yes	497(91.4) 47(8.6)	253(93.0) 19(7.0)	Ref. 0.9(0.6–1.3)	Ref. 0.8(0.5–1.2)
Postpartum hemorrhage	No Yes	531(97.6) 13(2.4)	245(90.1) 27(9.9)	Ref. 2.1(1.7–2.7) **	Ref. 2.1(1.6–2.7) **
Preeclampsia	No Yes	501(92.1) 43(7.9)	247(90.8) 25(9.2)	Ref. 1.1(0.8–1.6)	Ref. 0.97 (0.7–1.4)
Eclampsia	No Yes	531(97.6) 13(2.4)	261(95.9) 11(4.1)	Ref. 1.4(0.9–2.2)	Ref. 1.1(0.6–1.9)

^**^statistically significant p < 0.001 & ^*^statistically significant p < 0.05.

### Risk of adverse maternal outcomes among grand multi-parous women

According to multivariate Poisson regression modeling, the risk of postpartum hemorrhage was two times higher in grand multiparity (adjusted relative risk; (ARR) = 2.1;95 % CI: 1.6 ,2.7). Grand multiparity was associated with a higher probability of malpresentation (ARR = 1.3; 95 % CI: 1.01, 1.7) than multiparity. The model was adjusted for maternal age, religion, ethnicity, household wealth status, respondents' education, husbands' education, and occupation, as well as a history of preterm birth, stillbirth, unplanned pregnancy, undernutrition, and the newborn's sex (Table 3).

## Discussion

The incidence of adverse maternal outcomes of grand multiparous women was compared to those of multiparous women in this hospital-based prospective analysis. The cumulative frequency of negative maternal outcomes was 39.9% overall (95%CI: 36.6, 43.4%). Separately, poor maternal outcomes were found in 47.1% of grand multiparous women (95%CI: 41.0–53.2) and 36.3%of multiparous women (95% CI:32.3–40.6). In this study, grand multiparous women have a higher incidence of maternal outcomes than multiparous women (malpresentation, postpartum hemorrhage, and total days stay in the hospital, *P* < 0.05). While cesarean delivery, induction/augmentation, preeclampsia, and preterm membrane rupture were all more common in multiparous women, the differences were not statistically significant. The incidence of adverse maternal outcomes was higher among grand multiparas than in multiparas, according to the findings of this study. The findings are in line with a prior study conducted in Ethiopia (6). However, the result was higher than that of an Indian study (20). The possible explanation might be due to high standards and quality maternity services at all levels of health facilities in India. In the context of appropriate health-seeking behavior, studies suggest that adequate antenatal care and routine follow-up reduce the chance of birth complications (16). Similarly, the adoption of a contemporary healthcare system with favorable socio-economic and prenatal access could reduce the frequency of adverse maternal outcomes (50).

In this prospective cohort study, grand multiparous women had a higher risk of adverse maternal outcomes, according to our findings. The finding is comparable with the study done in Iraq (25). In contrast to the prior finding in Ethiopia, there was no significant difference between adverse maternal outcomes among multiparas and grand multiparas (6). This may be due to differences in sample size, study design, and analytic method.

Postpartum hemorrhage was found to be significantly higher among grand multiparous women than in the control group in this study. This is comparable to the Tanzanian study (22), two states in Nigeria (10, 51), and northern Pakistan (52). In this connection, our study showed that grand multiparas are at a significantly increased risk of developing postpartum hemorrhage compared with multiparas. A study conducted in Eastern Saudi Arabia came to the same conclusion (53). This is inconsistent with the study done by Muniro et al. (22) among Tanzanian women, Iraq (35), and Cameroon (1). This might be attributed to the variance in the study design approach differences, sample size, the standard of available medical services, and parity-related references in these studies. Likewise, more research is needed to build a body of knowledge about whether grand multiparity is a true risk factor for postpartum bleeding.

The current study discovered that grand multiparous women have a much higher rate of malpresentation than multiparous women. This finding is comparable to a study conducted in Pakistan study (54), India (55), Bangladesh (56), and northern Pakistan (52). However, in previous studies conducted in Nigeria (10), there were no significant changes in malpresentation between the exposed and non-exposed groups, according to the findings. In this study, it was observed that grand multiparous women have a considerably higher risk of malpresentation than multiparous women. These findings corroborate that of a study conducted in Kano, Nigeria (57), Tanzania (7), and Eastern Saudi Arabia (53). Grand multiparas are prone to numerous fetal malpresentation (58). Baby size and inborn abnormalities that decrease the tone of abdominal muscles, pendulous belly bllies, and room spaces in the womb are usually the contributing factors (59). Failure to predict and manage this malpresentation directly affects the outcome of labor with increased maternal morbidity and mortality (46).

This study revealed that there was no significant association between grand multiparity and cesarean section, preeclampsia/eclampsia, PROM, and induction/augmentation of labor, *p* > 0.05. This agreed with other findings conducted in Pakistan (30). However, in another study, induction of labor was significantly higher in multiparity than in grand multiparity (10). Other previously conducted research findings showed that grand multiparas were significantly associated with increased incidence of cesarean section and pregnancy-induced hypertension (11).

The methodological design of this study is one of its strengths since it proves causation between adverse maternal outcomes and grand multiparity. Furthermore, we used a rather large sample size to increase power and allow us to correct for confounders as well as draw highly precise conclusions in similar circumstances. Despite its strengths, some limitations should be considered when interpreting the results of this study. Because this study was conducted in a hospital, the findings may only apply to our study setting and others comparable to it. Some variables were not used to assess risk factors for adverse maternal outcomes due to the likelihood of confounding effects. This is prospective cohort research, but there is only a brief period of follow-up following delivery. Also, some non-random selection could be a source of bias.

## Conclusions

Grand multiparity is a high risk for adverse maternal outcomes during pregnancy and childbirth. These adverse maternal outcomes include postpartum hemorrhage and malpresentation observed in this study. The risk factor can be effectively decreased with good antenatal care and delivery by trained health providers. In low-resource settings, we recommend community health education and the provision of accessible and effective contraceptive services utilization as one strategy to prevent women from not getting high parity. It should also increase awareness of the adverse maternal outcome among grand multiparity during pregnancy on obstetric performance should be prioritized.

## Data availability statement

The raw data supporting the conclusions of this article will be made available by the authors, without undue reservation.

## Ethics statement

The studies involving human participants were reviewed and approved by Pan Africa University Life and Earth Sciences Institute, the University of Ibadan, and the University of Ibadan/University College Hospital, Ibadan Ethics Committee (Ethics committee assigned number /EC/20/0439). The patients/participants provided their written informed consent to participate in this study.

## Author contributions

All authors contributed equally to the work reported, whether in the conception, study design, execution, data acquisition, analysis, and interpretation, or all of these areas, participated in the drafting, revising, or critical review of the article, gave final approval of the version to be published, agreed on the journal to which the article was submitted, and agreed to be accountable for all aspects of the work.

## Funding

The Pan African University of Life and Earth Sciences Institute, Pan African University, and the African Union provided support for this study. The sponsoring organization played no part in the study's design, data collection, analysis, interpretation, or paper writing. The author's job was to do just that. Funders, on the other hand, did not pay a publication fee.

## Conflict of interest

The authors declare that the research was conducted in the absence of any commercial or financial relationships that could be construed as a potential conflict of interest.

## Publisher's note

All claims expressed in this article are solely those of the authors and do not necessarily represent those of their affiliated organizations, or those of the publisher, the editors and the reviewers. Any product that may be evaluated in this article, or claim that may be made by its manufacturer, is not guaranteed or endorsed by the publisher.
